# Smartband-based smoking detection and real-time brief mindfulness intervention: findings from a feasibility clinical trial

**DOI:** 10.1080/07853890.2024.2352803

**Published:** 2024-06-01

**Authors:** Mark Horvath, Brian Pittman, Stephanie S. O’Malley, Aurora Grutman, Nashmia Khan, Ralitza Gueorguieva, Judson A. Brewer, Kathleen A. Garrison

**Affiliations:** a Department of Psychiatry, Yale School of Medicine, New Haven, CT, USA; b Department of Biostatistics, Yale School of Public Health, New Haven, CT, USA; c Department of Behavioral and Social Sciences, Brown University School of Public Health, Providence, RI, USA

**Keywords:** Wearable, smartband, digital health, mindfulness, smoking

## Abstract

**Background:**

Smartbands can be used to detect cigarette smoking and deliver real time smoking interventions. Brief mindfulness interventions have been found to reduce smoking.

**Objective:**

This single arm feasibility trial used a smartband to detect smoking and deliver brief mindfulness exercises.

**Methods:**

Daily smokers who were motivated to reduce their smoking wore a smartband for 60 days. For 21 days, the smartband monitored, detected and notified the user of smoking in real time. After 21 days, a ‘mindful smoking’ exercise was triggered by detected smoking. After 28 days, a ‘RAIN’ (recognize, allow, investigate, nonidentify) exercise was delivered to predicted smoking. Participants received mindfulness exercises by text message and online mindfulness training. Feasibility measures included treatment fidelity, adherence and acceptability.

**Results:**

Participants (N=155) were 54% female, 76% white non-Hispanic, and treatment starters (n=115) were analyzed. Treatment fidelity cutoffs were met, including for detecting smoking and delivering mindfulness exercises. Adherence was mixed, including moderate smartband use and low completion of mindfulness exercises. Acceptability was mixed, including high helpfulness ratings and mixed user experiences data. Retention of treatment starters was high (81.9%).

**Conclusions:**

Findings demonstrate the feasibility of using a smartband to track smoking and deliver quit smoking interventions contingent on smoking.

## Introduction

Cigarette smoking is the leading cause of preventable disease, disability and death worldwide and costs over $1.4 trillion annually in direct healthcare costs and lost productivity [1,2]. Although 68% of individuals who smoke want to quit, only 7% achieve this annually [3], with less than one third using evidence-based treatments to support quit attempts [4]. Digital technology such as smartphone apps offer new ways to get effective treatment into the hands of the individual who smokes. Most U.S. adults (85%) own a smartphone [5], including 80% of adults who are motivated to quit smoking [6], and smartphone ownership is comparable across demographic groups [5]. Smartphones have advantages for delivering quit smoking interventions including scalability, low-cost and high-reach [7]. Daily use of smartphones is intensive: U.S. adults spend on average 4 h and 25 min on their phones each day and check their phones 144 times per day. Most (89%) check their phones within 10 min of waking up and 75% check their phones within 5 min of receiving a notification [8]. This dramatically increases the points of care from the clinic to nearly any time and place an individual needs support [9].

Evidence for the feasibility and efficacy of smartphone apps to deliver smoking cessation treatment is mixed [10,11]. A recent review found low certainty evidence for quit smoking apps compared with lower intensity smoking cessation support across four clinical trials meeting inclusion criteria [12]. However, a more recent trial found that an app for acceptance and commitment therapy for smoking cessation was more efficacious than a comparator quit smoking app [10]. The content [13,14], quality [15] and research [16] of quit smoking apps have been reviewed, identifying several limitations, including a lack of delivery of evidence-based treatment and few randomized clinical trials. Most apps were found to provide simplistic tools such as manual smoking trackers and calculators and most fall short of adhering to clinical practice guidelines [16,17].

Notably, few quit smoking apps utilize smartwatches or smartbands to help individuals quit smoking (for a broader review of wearables and smoking see [18]). Smartwatches (i.e. extend smartphone) and smartbands (i.e. separate wearable) offer new ways to bolster and integrate with effective quit smoking treatments and are being used to track health behavior more widely. One in three U.S. adults now wear a smartwatch or wearable health tracker; wearable technology use jumped 100% from 2016 to 2019; and again, use rates are comparable across demographic groups [19,20]. The projected growth of the wearables market, valued at $61.3 billion in 2022 and expected to grow 14% from 2023 to 2030 [19,21], suggests that quit smoking programs that exploit this technology will have wide population reach. Smartbands/watches have been recently developed to utilize data from wrist sensors to detect the hand-to-mouth gestures of smoking a cigarette and differentiate smoking from other similar hand gestures. Several smartbands/watches have been initially validated to detect cigarette smoking in laboratory [22] and real-world settings [18,23,24], including using proprietary sensors [25,26] or off-the-shelf smartbands/watches [23,24,27]. Several smartbands/watches have been found to accurately and precisely detect smoking in the real world [23–25,27,28], to be acceptable to individuals who smoke cigarettes [29] and to have promising effects on smoking [27,29].

Using smartbands/watches to monitor and detect smoking may be advantageous due to their availability, low cost, popularity and the convenience and completeness of data collection [30]. Measuring smoking by smartband/watch requires little to no action by the user and is thus considered highly unobtrusive [30]. Smartband/watch connectivity is also continuous, enabling real time collection and use of smoking data. Importantly, using smartbands/watches for automatic, passive, continuous tracking of smoking reduces the burden on the individual to track and self-report their own smoking. Most quit smoking apps require users to track their own smoking [17], which can be unreliable [31] and result in low treatment engagement [32]. Studies in which participants are asked to record each cigarette smoked showed low compliance related to fatigue, forgetting and lack of real-time awareness of the behavior [33,34]. Several smartbands/watches will additionally notify users of detected smoking in real time (e.g. *via* smartband app notification and vibration of the smartband/watch) [23,25,27]. Notification of smoking may bring awareness to automatic smoking behavior (e.g. lighting a cigarette without thinking about it) [35], which is a central feature of nicotine dependence [36]. Only a few studies have progressed beyond validating smartbands/watches for smoking detection to testing their use to deliver interventions for smoking [28,29,37].

Mindfulness-based interventions may be well-suited to deliver by smartband/watch because mindfulness is thought to target momentary risk factors of smoking such as craving [38]. Recent work suggests that mindfulness-based interventions are effective for smoking cessation [39], although evidence is mixed [40]. Mindfulness is typically considered to involve paying attention in the present moment, on purpose and nonjudgmentally [41]. Mindfulness-based interventions may help individuals who smoke learn to pay attention to cravings as they arise and work mindfully with cravings, rather than to react by smoking [38]. In a recent study, a mindfulness-based intervention for smoking was found to be feasible to deliver by smartphone app and lessened the association between craving and smoking [11].

Building on this initial work, the current trial tested the feasibility of using a smartband to monitor, detect and notify individuals of smoking events in real time and to deliver brief mindfulness exercises either triggered by smartband-detected smoking events or delivered to predicted smoking events based on tracking smoking data. This single arm clinical trial measured treatment fidelity, adherence and acceptability of the approach. The main purpose of the feasibility trial was to demonstrate using a smartband to track smoking and deliver smoking interventions in real time.

## Methods

### Overview

A feasibility clinical trial was conducted (NCT03995225). The trial protocol was reported previously [42]. Ethical approval for the study was obtained from the Yale University Institutional Review Board (IRB Protocol #2000025082). All participants provided online written informed consent. The trial was fully remote. The trial tested the primary outcome measures of treatment fidelity, adherence and acceptability and secondary outcome measures of cigarettes per day (CPD) and smoking abstinence rates. Table S1 presents the participant flow. Participants wore a smartband for 60 days to monitor and detect smoking and notify the user of smoking events in real time. They set a quit date at 30 days. From 21 days, a ‘mindful smoking’ exercise was triggered by smartband-detected smoking events. From 28 days, a ‘RAIN’ exercise (recognize, allow, investigate, non-identify) was delivered to predicted smoking events, based on the first 21 days of tracking smoking data. (This period was used to test how much tracking smoking data would be needed to predict smoking; findings not presented here). The brief mindfulness exercises were adapted from the Craving to Quit app [11]. Additional mindfulness training adapted from Craving to Quit was provided online at 21 and 28 days. Study surveys were administered online at baseline, 21, 28 and 60 days. Participants were compensated for onboarding, surveys, wearing the smartband daily and a study completion bonus and were paid at the end of the study, after returning the study equipment.

### Participants

Participants were eligible if they were 18 years or older, smoked at least 5 cigarettes per day (CPD) for at least 2 years, owned an iPhone or Android phone, were fluent in English as intervention content was only available in English at study onset and were motivated to reduce their smoking as indicated by a score of at least 4 out of 5 on one item of the Action subscale of the Readiness to Change Questionnaire, ‘I am trying to smoke less than I used to’ from 1 (strongly disagree) to 5 (strongly agree) [43].

### Recruitment

Recruitment was conducted nationally across the U.S. between 1 May 2021 and 15 December 2021 using Facebook advertisements. Advertisements linked to a study website and short screening survey hosted on Yale Secure Qualtrics Survey Tool. Qualtrics ‘prevent ballot box stuffing’ was used to prevent individuals from taking the survey more than once, which was also confirmed by IP address. If eligible, individuals were directed to an online informed consent form and then asked for their contact information (to ship the study smartband). Those who provided their contact information and informed consent were asked to respond by text message to confirm their interest in enrolling in the study. Those who confirmed their interest were shipped a study smartband and scheduled a video call with a researcher for onboarding to the study technology.

### Study smartband

The study smartband was developed by Somatix, Inc [27,44]. The smartband uses a machine-learning algorithm to accurately identify the distinct hand-to-mouth gestures that are characteristic of smoking a cigarette and differentiate these from other similar hand gestures specific to each individual, based on raw data collected from accelerometer and gyroscope sensors, which are subsequently subjected to data stabilization and noise filtering procedures. Through rigorous analysis, the algorithm determines the specific movements performed by the individual that signify cigarette smoking. Upon detecting a smoking event (typically after three to four puffs), the smartband vibrates and the user receives a notification on their smartphone to confirm or deny smoking *via* the corresponding smartphone app.

### Onboarding

The onboarding session was used to guide the participant in setting up their smartband and corresponding smartphone app, including how to charge and wear the smartband (i.e. on the hand used to smoke and how it should be oriented on the wrist), download the app and pair the smartband and app, configure app settings (e.g. allow Bluetooth to pair with smartband, allow app notifications, let app run in the background for continuous smoking monitoring), login to the app using a study PIN, take a quick tour of app features (e.g. check current status of smartband battery and pairing with smartband) and troubleshooting tips; and to review the study procedures. After onboarding, participants were sent an email reminding them of the study procedures and instructing them to visit smokefree.gov medications webpage because ‘medications can double your chances of quitting smoking for good’. They were also sent a baseline survey link by text message, to begin the study. Individuals were considered enrolled in the trial after completing onboarding.

### Intervention

Participants received a smartband and app and took part in a 60-day intervention. For the first 21 days, they were asked to wear the smartband on the hand they used to smoke for 12 h per day. The smartband automatically and continuously tracked their smoking. When a smoking event was detected, the smartband vibrated and a notification was sent *via* the smartphone app asking them to confirm or deny smoking, to bring awareness to their smoking. If they smoked and it was not detected by the smartband, they were asked to manually report smoking on the app (by hitting ‘+’ under ‘Cigarettes Smoked’ on the homepage of the app), which they could do at any time. They were instructed to smoke as much or as little as they liked and to set a quit date at 30 days. They were automatically sent a reminder notification from the app if they wore the smartband <12 h on the previous day. They were sent text message (after 48 h) and email (after 72 h) reminders if they wore the smartband <12 h per day. This 21-day period additionally provided tracking smoking data used to predict smoking events later in the intervention.

At 21 days, participants were sent a 21-day survey link by text message. At the end of the survey, they took part in a brief online mindfulness training [11], using video and animation to introduce mindfulness, habit formation and a guided mindful smoking exercise. The mindful smoking exercise brings awareness to the present moment effects of smoking – the physical sensations, emotions and thoughts that arise from smoking [38,45] – to begin a process of disenchantment with smoking [38,46]. For the following week, the smartband continued to monitor and detect smoking, notify them of smoking events in real time and ask them to confirm or deny smoking and manually report any missed smoking events. In addition, any time a smoking event was detected by the smartband, the mindful smoking exercise was delivered by text message. The mindful smoking exercise was a 2 min audio with captions, after which they were asked to rate, ‘How helpful did you find this exercise?’ (visual analog scale [VAS] from ‘not at all helpful’ to ‘very helpful’.)

At 28 days, participants were sent a 28-day survey link by text message. Again the survey was followed by a brief online mindfulness training, using video and animation to introduce the concepts of craving and urge surfing and a guided RAIN exercise: Recognize, Allow, Investigate, Nonidentification [38], to work mindfully with cravings rather than to react by smoking [11,47]. For the following month (after their quit date), the smartband continued to monitor and detect smoking, notify them of smoking events in real time and ask them to confirm or deny smoking and manually report any missed smoking events. Any time a smoking event was detected by the smartband, the mindful smoking exercise was delivered by text message. In addition, they were texted the RAIN exercise to predicted smoking events. The RAIN exercise was a 2 min audio with captions, before and after which they were asked to rate, ‘How much are you craving a cigarette right now?’ (VAS, ‘not at all’ to ‘very much’) and ‘How are you feeling right now?’ (VAS, ‘very bad’ to ‘very good’, i.e. mood) and after which they were also asked to rate, ‘How helpful did you find this exercise?’ (VAS, ‘not at all helpful’ to ‘very helpful’) and ‘How was the timing of this exercise?’ (VAS, ‘too early’ to ‘too late’). Smoking events were predicted for each individual based on their tracking smoking data from the first 21 days of the study. To do this, a basic prediction algorithm was executed every 5 min based on smoking timeseries data. If >1 smoking event was detected within a 15-min window of the current time on any day during the 21-day period, the prediction was considered true. In other words, if a participant had smoked at that time during the first 21 days, it was considered a moment of heightened risk. To avoid sending too many RAIN exercises, a text cooldown period was implemented, such that RAIN was not sent twice within 45 min. These settings were established based on an initial pilot study involving eight participants who met the same inclusion criteria and completed the study procedures and were not included in the current analysis. The overall goal was to deliver quit smoking support while minimizing the burden of text messages on participants.

Throughout the intervention, participants could skip the mindfulness exercises by ignoring text messages. They could also access the mindfulness exercises any time by clicking the link in their text messages. Completion of mindfulness exercises was time stamped including onset and offset times. To encourage engagement, daily text messages were sent reporting the number of mindfulness exercises they completed on the previous day or reminding them to use the mindfulness exercises if none were completed. Participants could also opt out of text messages and opt back in at any point.

At 60 days, participants were sent an end of treatment survey link by text message. At the end of the survey, participants who reported one week point prevalence abstinence from smoking were shipped a saliva cotinine test with instructions and recorded and securely uploaded a video during which they used the saliva test and displayed the output. Once a participant completed the end of treatment survey and returned any study equipment, they were considered to have completed the study. Participants were asked to return the study smartband due to equipment limitations at the time.

## Measurements

### Demographics

PhenX Toolkit (https://www.phenxtoolkit.org/) protocols were used to assess demographics including Sex Assigned at Birth, Gender Identity, Ethnicity and Race, Current Age, Educational Attainment and Annual Family Income.

### Treatment fidelity

Treatment fidelity was assessed with the following measures. (1) *Accurate detection of smoking events* was measured as the percent of smoking events correctly detected (number confirmed/[number confirmed + denied]) and percent of false alarms (i.e. smartband classifies another hand gesture as smoking) and was measured across the 60-day intervention. Based on an earlier pilot study, we expected >80% detection and negligible percent of false alarms [27]. (2) *Delivery of the mindful smoking exercise triggered by smoking events* was measured as the percent of mindful smoking events correctly triggered by smoking events (1 – [number denied/(number confirmed + denied + pending + manually reported)]) (where pending indicates smoking events detected by the smartband but neither confirmed nor denied by the participant) and percent of false alarms (i.e. mindful smoking triggered by smartband false alarm) and was measured from day 21 to end of treatment. Again, we expected >80% detection and negligible percent of false alarms. (3) *Delivery of the RAIN exercise to predicted smoking events* was measured as mean craving rated prior to completing RAIN and mean timeliness rated after completing RAIN, each measured from day 28 to end of treatment. Treatment fidelity was determined by participants rating timeliness as ‘about right’ or better (i.e. mid-range of the VAS) and craving as moderate or higher (i.e. equal to or greater than the VAS midpoint) on 75% of RAIN exercises. The VAS were coded as 0–10. Additionally, we explored mood by calculating mean mood rated prior to completing RAIN, measured from day 28 to end of treatment. We also report the percent of participants who completed at least one RAIN exercise who rated timeliness as ‘about right’ or better, craving as moderate or higher and mood as moderate or lower. Finally, we explored changes in craving and mood pre- to post-RAIN and did not have feasibility cutoffs for these measures.

### Adherence

Adherence to the smartband was measured as the percentage of days participants wore the smartband for ≥12 waking hours and percent of smoking notifications responded to ([number confirmed + denied]/[number confirmed + denied + pending]), across the 60-day intervention. Adherence to the mindful smoking exercise was measured as percent of mindful smoking exercises completed of those sent from day 21 to end of treatment. Adherence to the RAIN exercise was measured as percent of RAIN exercises completed of those sent from day 28 to end of treatment. Mindfulness exercises were considered completed if participants listened to the 2 min audio and answered the post-exercise ratings (e.g. helpfulness). We predicted 80% of mindfulness exercises would be completed for an individual. An additional measure related to adherence was asked at 60 days, ‘Did you complete [mindful smoking/RAIN] exercises on your own, without clicking the link to the guided exercise?’ (never/always).

### Acceptability

Acceptability was measured as mean helpfulness rating for the mindful smoking exercise, measured from day 21 to end of treatment and for the RAIN exercise, measured from day 28 to end of treatment. Acceptability was determined by participants rating helpfulness as moderate or higher (i.e. equal to or greater than the VAS midpoint) 75% of the time. We also report the percent of participants who completed at least one mindfulness exercise or at least one RAIN exercise who rated helpfulness as moderate or higher.

Acceptability was additionally evaluated using a user experiences questionnaire developed for this trial and administered as part of the surveys at onboarding, 21, 28 and 60 days, which included the following five-point Likert items. At onboarding, participants were asked to rate, ‘The instructions for getting started with my smartband were (easy/difficult) to follow’. At 21 days, participants were asked to rate, ‘I have been finding it (very easy/very difficult) to wear my smartband every day’, ‘It has been helpful to be notified of my smoking events’ (strongly agree/strongly disagree), ‘I like being notified of my smoking events’ (strongly agree/strongly disagree) and ‘Being notified of my smoking events has helped me pay attention to my smoking’ (strongly agree/strongly disagree). At 28 days, participants were asked to rate, ‘The mindful smoking exercise has been helpful’ (strongly agree/strongly disagree), ‘I like the mindful smoking exercise’ (not at all/extremely) and ‘The mindful smoking exercise has helped me pay attention to my smoking’ (strongly agree/strongly disagree). At 60 days, participants were asked to rate, ‘The RAIN exercise has been helpful (strongly agree/strongly disagree), ‘I like the RAIN exercise’ (not at all/extremely), ‘The RAIN exercise has helped me cope with craving to smoke’ (strongly agree/strongly disagree) and ‘The RAIN exercise was sent at the right time’ (never/always). Additionally, they were asked, ‘I have been finding it (very easy/very difficult) to keep my smartband charged’, ‘I have been finding it (very easy/very difficult) to keep my smartband paired to my smartphone’, and, ‘On each day that the [mindful smoking/RAIN] exercise was sent, it was sent…’ (far less often/far more often than I would like). For ratings, acceptability was determined as 75% of participants rating a given item as moderate or better.

Additional standard scales were included at the 60-day survey, adapted for the trial as indicated. The User Burden Scale [UBS; 48] is a 20-item scale measuring user burden in computing systems. Items are scored from 0 (‘never/not a lot’) to 4 (‘all of the time/extremely’) and items are summed for a total score, range 0–80, with higher scores indicating higher user burden. In addition to the total score, we report subscale scores for the burden of ‘difficulty of use’, (range 0–16) ‘time and social’, (range 0–16) ‘mental and emotional’ (range 0–16) and ‘privacy’ (range 0–12). We omitted the subscales ‘physical’ and ‘financial’ burden for lack of relevance to the intervention. Another approach would have been to have participants complete all subscales and indicate ‘0’ to indicate ‘no burden’ for the irrelevant subscales [48]. Total score for the UBS with the included subscales was range 0–60. From the Mobile Application Rating Scale [MARS] [49] we included Section F, which assesses the perceived impact of the intervention on the user’s knowledge, attitudes, intentions to change and likelihood of actual change in the target health behavior (here, smoking), using six-items, each rated on a five-point Likert scale from 1 (‘strongly disagree’) to 6 (‘strongly agree’), scored as the mean, with higher scores indicating greater perceived impact on smoking. We also included the Acceptability of Intervention Measure (AIM), Intervention Appropriateness Measure (IAM) and Feasibility of Intervention Measure (FIM) [50] measures often considered ‘leading indicators’ of implementation success [51]. Each measure includes four items rated on a five-point Likert scale from 1 (‘completely disagree’) to 4 (‘completely agree’), scored by averaging (range 1–4), with higher scores indicating greater acceptability, appropriateness, or feasibility. Finally, exit interviews were conducted with *n* = 10 participants and these methods and qualitative findings are summarized in the supplement.

### Secondary measures

The following additional secondary outcomes were measured at baseline and 60 days: CPD; Fagerstrom Test for Nicotine Dependence [FTND] [52] total score range 0–10 with higher scores indicating higher nicotine dependence; Minnesota Nicotine Withdrawal Scale [MNWS] [53] mean score range 1–4 with higher score indicating greater withdrawal; Five Facet Mindfulness Questionnaire short form [FFMQ] [54] which includes 24 items rated 1–5, total score range 24–120, with higher scores indicating higher mindfulness; and at 60 days: one-week point-prevalence abstinence and prolonged abstinence with lapses (≤5 cigarettes since quit date) [55]. Those who reported one-week point-prevalence abstinence were asked to provide biochemical verification of abstinence as described above.

## Statistical analysis

Demographic variables and primary measures of treatment fidelity, adherence and acceptability were summarized descriptively by estimating percentages, means, standard deviation (SD), medians, and ranges. All available data for each participant was used, regardless of missing data. Secondary measures were also summarized using descriptive statistics. In addition, linear mixed models, which utilize all available data from each subject, were performed to evaluate within-subject changes from baseline to end of treatment in CPD, FTND, MNWS, and FFMQ. Least-square (LS) means and standard errors (SEM) were estimated from the model. All analyses were conducted using SAS, version 9.4 (SAS Institute Inc., Cary, NC, USA). The statistical analysis plan was preregistered (NCT03995225).

## Results

### Participant characteristics

**Figure 1.**
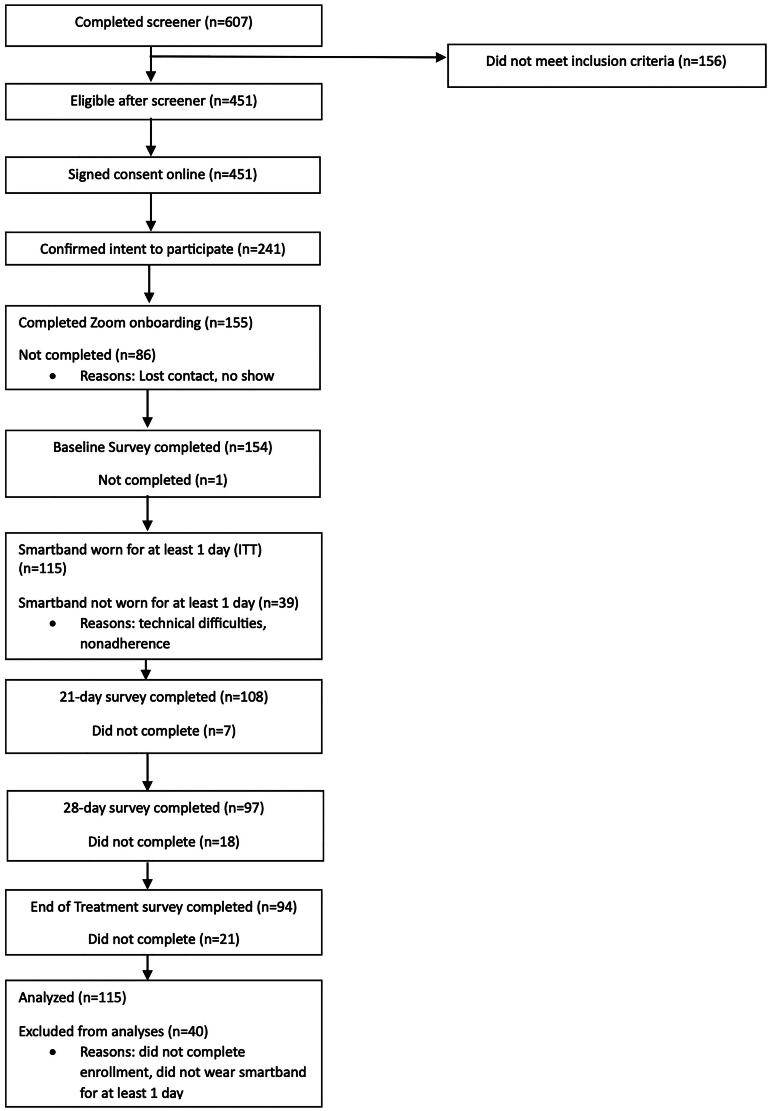
CONSORT diagram.

Participant demographics for the full intent to treat (ITT) sample (*n* = 155 completed enrollment) are reported in Table 1. Herein, results are reported for a modified intent to treat (ITT) sample (as previously [11]) among enrolled subjects who wore the smartband for at least one day (treatment starters; *n* = 115; Figure 1). We note that results were largely indistinguishable between treatment starters and the full ITT sample, given that most findings relate to smartband use (see supplement for available findings for the full ITT sample). For modified ITT, retention was 96.6% at 21 days, 87.1% at 28 days and 81.9% at 60 days. Additional information about participants lost during enrollment is provided in the supplement.

**Table 1.** Participant characteristics (total *n* = 155).

Demographics	n	%	Mean	SD	Range
Age (years)			46.1	10.4	22–76
Female/woman	84	54			
Race/ethnicity					
Non-Hispanic White	118	76.1			
Hispanic White	3	1.9			
Black or African American	6	3.9			
Asian	5	3.2			
Some other race/multiracial	23	14.8			
Education					
GED/equivalent or high school or less	32	20.7			
Education (years)			16.8	2.2	11–22
Annual family income					
<$20,000	22	14.2			
$20,000–75,000	82	52.9			
>$75,000	47	30.3			
Refused/don’t know	4	0.3			

### Treatment fidelity

Treatment fidelity measures are reported in Table 2. 90% of smoking events were accurately detected by the smartband. Mindful smoking was correctly triggered (i.e. by smartband detected or manually reported smoking events) 96% of the time. 83% of participants rated the delivery of RAIN (i.e. to predicted smoking events) as timely. Across all RAIN exercises, 75% were rated timely. Craving ratings were just under feasibility cutoffs, with 70% of participants rating at least moderate craving when RAIN was delivered. Across all RAIN exercises, craving was rated at least moderate 64% of the time.

**Table 2.** Treatment fidelity.

Intervention delivery	n^a^	Mean	SD	
Percent smoking events correctly detected	111	90.2	17.5	
Percent false alarms	111	9.8	17.5	
Percent mindful smoking correctly triggered	114	95.6	10.9	
Participant ratings	Total	n	%	
Participants rating RAIN as moderately or more timely	84	70	83.3	
Participants rating moderate or higher craving at RAIN	94	66	70.2	
Participants rating moderate or lower mood at RAIN	94	17	18.1	
Ratings across all exercises in real time	Total	%	Mean^b^	SD
RAIN rated as moderately or more timely	3781	74.8	5.66	1.99
Craving rated as moderate or higher at RAIN	3846	64.2	5.61	2.83
Mood rated as moderate or lower at RAIN	3846	28.0	6.74	2.23

^a^*n* = 111 responded to at least one smoking notification; *n* = 114 triggered at least one mindful smoking exercise by smoking detection or manual report; *n* = 84 completed one RAIN exercise; *n* = 94 initiated one RAIN exercise; 3781 RAIN exercises were completed; 3846 RAIN exercises were initiated.

^b^Rated out of 10.

Among exploratory outcomes for treatment fidelity, mood ratings did not meet feasibility cutoffs, with 18% of participants rating mood when RAIN was delivered as moderate or lower. Across all RAIN exercises, mood was rated as moderate or lower 28% of the time. Finally, linear mixed models showed that there was a decrease in craving (F(1,83)=187.0, *p*<.0001; LS mean change= −0.58 ± 0.04 [SEM]) and mood (F(1,83)=6.51, *p*<.02; LS mean change = 0.09 ± 0.04 [SEM]) pre- to post-RAIN.

### Adherence

Adherence measures are reported in Table 3. Participants wore the smartband on 70% of treatment days (10 ± 3 h per day) and for ≥12 h per day on 41% of treatment days. Participants responded to (confirmed/denied) 98% of smoking events. Participants completed 40% of mindful smoking exercises (0.9 ± 1.4 out of 2.7 ± 3.4 sent per day) and 8% of RAIN exercises (1.3 ± 2.4 out of 16.6 ± 5.6). Of note, once initiated (i.e. clicked text message link), most mindfulness exercises were completed. Of 2495 mindful smoking exercises initiated, 100% were completed, and of 3846 RAIN exercises initiated, 98.3% were completed. Additionally, the average number of RAIN exercises sent per day (16.6 ± 5.6) was consistent with average baseline smoking (18.6 ± 8.7 CPD, see ‘Secondary measures’ below). Finally, many participants reported completing mindful smoking (60%) and RAIN (64%) on their own at least ‘sometimes’ or more often, either because they could not access (18%) or use (33%) their phone, they felt confident to complete the exercise on their own (57%), or other reasons (6%).

**Table 3.** Adherence.

*Smartband adherence*	n^a^	Mean	SD	Median	Range
% days wearing smartband (of 60 days)	115	69.6	30.5	80	6.7–100
% days wearing smartband ≥12 h (of 60 days)	115	40.8	29.7	36.7	1.7–98.3
% smoking notifications responded to	111	98.2	4.0	100	70.6–100
	n	Mean	SD	Median	Range
Hours per day	115	10.1	3.1	10.9	1.5–15
Mindfulness exercise adherence	n	%	SD	Range	
Mindful smoking completed	75	40.3	31	0.9–125^b^	
RAIN completed	94	7.9	13.9	0–88.1	
	Total	n	%		
Participants completing mindful smoking on own	95	60	61		
Participants completing RAIN on own	95	64	65		

^a^*n* = 115 wore the smartband at least 1 day; *n* = 111 responded to at least one smoking notification; *n* = 75 completed at least one mindful smoking exercise; *n* = 94 initiated at least one RAIN exercise, *n* = 95 completed 60-day survey.

^b^*n* = 2 participants used the text link to complete more mindful smoking exercises than they were sent.

### Acceptability

Acceptability measures are reported in Table 4. 75% of participants rated mindful smoking as helpful. Across all mindful smoking exercises, 81% were rated as helpful. 76% of participants rated RAIN as helpful. Across all RAIN exercises, 82% were rated as helpful.

**Table 4.** Acceptability.

Participant ratings in the moment	n^a^	%		
Participants rating mindful smoking as moderately or more helpful	75	74.7		
Participants rating RAIN as moderately or more helpful	84	76.2		
Ratings across all exercises in the moment	n	%	Mean^b^	SD
Mindful smoking rated as moderately or more helpful	2495	80.6	6.6	3.1
RAIN rated as moderately or more helpful	3781	82	6.7	2.6
User experiences questions				
21 days survey	n	%	Mean^c^	SD
Onboarding instructions were easy to follow	112	98	1.5	0.7
Easy to wear smartband every day	112	90	1.8	1.0
Helpful to be notified of smoking	112	88	2.3	1.1
Liked being notified of smoking	112	93	2.1	1.0
Being notified of smoking helped them pay attention to their smoking	112	90	2.1	1.0
28 days survey				
Mindful smoking was helpful	101	79	2.7	1.1
Liked mindful smoking	101	58	2.8	1.1
Mindful smoking helped them pay attention to their smoking	101	92	2.2	0.9
60 days survey				
RAIN was helpful	95	75	2.9	1.2
Liked RAIN	95	52	2.5	1.8
RAIN helped them cope with craving to smoke	95	67	3.1	1.2
Mindful smoking sent too often	95	45	3.5	1.0
RAIN sent too often	95	62	3.8	1.0
Standardized questionnaires				
User Burden Scale	n	Mean^d^	SD	
Total score	95	7.5	7.8	
Difficulty of use	95	2.9	2.6	
Time and social burden	95	2.5	3.2	
Mental and emotional burden	95	1.3	2.5	
Privacy burden	95	0.9	1.5	
MARS F perceived impact on smoking behavior	95	4.1	0.7	
Acceptability of intervention measure (AIM)	95	3.8	0.9	
Intervention appropriateness measure (IAM)	95	3.9	0.9	
Feasibility of intervention measure (FIM)	95	4.0	0.8	

^a^*n* = 75 completed at least one mindful smoking exercise; *n* = 84 completed at least one RAIN exercise; 2495 mindful smoking exercises were completed; 3781 RAIN exercises were completed; *n* = 112 completed the 21-day survey; *n* = 101 completed the 28-day survey; *n* = 95 completed the 60-day survey.

^b^Mindfulness exercises were rated 1–10.

^c^User experiences items at each 21, 28 and 60-day survey were rated 1–5.

^d^User Burden Scale, total score range 0–60, subscale scores range 0–16 or 0–12; MARS: Mobile Application Rating Scale, Section F, mean score range 1–6; AIM/IAM/FIM, mean score range 1–4.

At 21 days, most participants found the smartband instructions easy to follow (98%) and the smartband easy to wear (90%). Most participants found the smoking notifications helpful (88%), particularly with helping them pay attention to their smoking (90%) and most liked the smoking notifications (93%). At 28 days, a majority of participants found mindful smoking helpful (79%), especially in drawing attention to their smoking (92%), although only a slim majority liked mindful smoking (58%). At 60 days, a majority of participants found RAIN helpful (75%), slightly fewer reported that RAIN helped with craving (67%); and a slim majority liked RAIN (52%). Most (79%) reported that it was easy to keep their smartband charged, although about half (52%) reported difficulties keeping their smartband paired to their smartphone. Finally, many participants reported that the mindful smoking (45%) and RAIN (62%) exercises were sent too often.

Additionally, at 60 days, participants rated low user burden (User Burden Scale total = 7.5 ± 7.8); high perceived impact on smoking behavior (MARS Section *F* = 4.1 ± 0.7) and high acceptability (AIM = 3.8 ± 0.9), appropriateness (IAM = 3.9 ± 0.9) and feasibility (FIM = 4.0 ± 0.8).

## Secondary measures

Secondary outcomes are reported in Table 5. Average CPD decreased from baseline (18.6 ± 8.7) to 60 days (F(1, 114)=55.2, *p*<.0001; LS mean change= −9.3 ± 1.3 [SEM]). One-week point-prevalence abstinence was reported by *n* = 14 (12.2% with missing data coded as smoking). Participation in biochemical verification of abstinence was low (*n* = 5, 36%) based in part on shipping delays due to COVID-19 (additionally, only *n* = 2 successfully verified abstinence, *n* = 2 were positive for cotinine, *n* = 1 was invalid). Prolonged abstinence with lapses (≤5 cigarettes since quit date [55]) was reported by *n* = 29 (25.2% with missing data coded as smoking). Finally, *n* = 21 (21.9%) reported using another approach to help them quit smoking cigarettes (varenicline, bupropion, nicotine replacement therapy (NRT), e-cigarette, or ‘other’).

**Table 5.** Secondary outcomes.

	Baseline			60 days		
	n	Mean	SD	n	Mean	SD
Cigarettes per day	115	18.6	8.7	95	9.4	12.8
Fagerstrom Test for Nicotine Dependence	115	5.4	2.3	95	5.4	2.3
Minnesota Nicotine Withdrawal Scale	115	1.3	0.6	95	3.7	2.5
Five Facets Mindfulness Questionnaire – short	115	80.2	10.3	95	83.3	10.8

There was a decrease in FTND from baseline (5.4 ± 2.3) to 60 days (F(1, 114)=65.8, *p*<.0001; LS mean change= −1.7 ± 0.2 [SEM]); an increase in MNWS from baseline (1.3 ± 0.6) to 60 days (F(1, 114)=143.0, *p*<.0001; LS mean change = 0.81 ± 0.07 [SEM]); and an increase in FFMQ from baseline (80.2 ± 10.3) to 60 days (F(1, 114)=10.1, *p* = .002; LS mean change = 3.1 ± 1.0 [SEM]).

Post-hoc, based on prior evidence that participation in brief mindfulness exercises impacts smoking cessation outcomes [47], we found that the percent of mindful smoking exercises completed was greater for participants who reported prolonged abstinence from smoking (*n* = 20, 57.6%) compared with those who did not (*n* = 51, 34.5%; *p*=.004; referring to participants who completed both the 60 day survey and mindful smoking). No associations were observed between completion of mindful smoking and change in CPD, or between completion of RAIN and prolonged abstinence or change in CPD.

## Discussion

This trial examined the feasibility of using a smartband to monitor and detect smoking, notify the individual of smoking events in real time and deliver brief mindfulness interventions triggered by smartband-detected smoking or delivered to predicted smoking events based on tracking smoking data. This single arm clinical trial measured treatment fidelity, adherence and acceptability. Retention of treatment starters in the study was very good. Treatment fidelity was largely established, and adherence and acceptability were mixed.

Treatment fidelity was largely established for the approach. The smartband was found to accurately detect smoking; mindful smoking exercises were successfully triggered by detected smoking; and RAIN exercises delivered to predicted smoking were reported to be timely. Although craving rated at the time RAIN was delivered did not meet feasibility cutoffs, there was a decrease in craving with RAIN. A main contribution of this trial is the demonstration that a smartband can be used to deliver quit smoking interventions contingent on smoking.

Adherence was less well established for the approach. Participants wore the smartband less than expected. Nevertheless, they wore the smartband on average 42 days, for 10 h per day and confirmed/denied almost all smartband-detected smoking events. Only limited research has used smartbands to track smoking in the real world, thus there is no benchmark for the level of smartband use needed to impact smoking. The only other reported feasibility study of a quit smoking smartwatch reported that 80% of participants used a corresponding smartphone app every day but did not report adherence (days/hours) to wearing the smartwatch [29]. In another feasibility trial, 63% of participants wore a set of four chest and wrist sensors for detecting smoking on 8 of 14 treatment days, falling somewhat short of an a priori feasibility cutoff of 80% [37]. In an earlier study using the same sensor suite, participants included in the analysis wore the sensors on average 14.6 h per day across a 14 day intervention [28]. In another domain, research using Fitbit to promote physical activity was found to use different metrics to report adherence to daily wear instructions, leading to difficulties in determining adherence cutoffs relevant to behavior change [56].

Adherence to mindfulness exercises (percent completed) was also low. Nevertheless, participants completed on average one mindful smoking and one RAIN exercise per day. One consideration is that the same mindfulness exercises were delivered numerous times and participants may have felt confident to complete the exercises on their own. Indeed, most participants reported completing the mindfulness exercises on their own at least sometimes, suggesting additional engagement beyond what was directly monitored. In the only comparable trial, 67% of participants completed at least 60% of mindfulness exercises delivered in response to sensor data indicating smoking risk, again somewhat short of an 80% cutoff [37]. In the current trial, adherence to mindful smoking was somewhat better than RAIN, possibly because participants already had their phone in hand when smoking, making it easier to complete the exercise when prompted. The main user feedback about the mindfulness exercises was that they were sent too often. Future studies might limit the number of exercises sent to participants daily to reduce this burden. Overall, findings suggest the need for strategies to improve adherence to interventions delivered based on smartband smoking data.

More generally, low real-world engagement is considered a main challenge in digital mental health [57], including wearables [58]. It has been argued that engagement cannot be measured as the number of times an individual accesses a digital intervention (e.g. logs onto an app), but needs to account for how much they engage with the intervention [59,60]. Yet, there is no standard definition of meaningful engagement in digital mental health interventions and no way to compare engagement across studies [60], with most studies reporting positive indicators for user engagement, but none using the same combination of criteria or thresholds [61]. Additionally, there is no consensus for how to deal with users who are not engaged but stay in the study [59]. It will be critical to determine in future studies what adherence and engagement thresholds are clinically meaningful to smoking cessation for intervention components such as daily smartband use and completion of mindfulness exercises.

Another consideration for adherence was technical difficulties with the smartband, which varied. Participants reported that it was easy to keep the smartband charged, but they had some problems keeping the smartband paired with their smartphone. Continuing to improve pairing between smartband and smartphone, both *via* technological improvements and by participant onboarding and technical support, would likely improve adherence to wearing the smartband, thereby allowing more accurate detection and prediction of smoking events to deliver mindfulness exercises and possibly improving adherence to completing the mindfulness exercises.

Despite reports of RAIN being sent more than participants would have liked, they were sent on average 17 RAIN exercises per day which corresponded with average baseline smoking of 19 CPD. This supports the feasibility of predicting smoking from smartband tracking smoking data. The algorithm used in this trial was rudimentary, based on basic smoking timeseries data. Other approaches have used other sensor data to predict smoking (or risk of smoking) such as geo-location [62], chest band and electrodes to measure electrocardiography and respiration as indicators of stress and smoking [28,37] and even context information from ecological momentary assessment [36,62]. Another quit smoking smartwatch uses more detailed smoking timeseries data (time of day, day of week, time related to eating, sleeping, driving) with location and social context (i.e. who is nearby based on repeated detection of Bluetooth static address) [29], although no data have been reported on prediction accuracy. Outside of smoking, passive sensing has included accelerometry, location, audio and usage data collected from smartphones to address health and wellbeing [63], suggesting additional potential measures. The current trial provides a basic demonstration that smartband tracking smoking data can be used to predict smoking, and future work is needed to develop the prediction algorithm.

Acceptability of the approach was also somewhat mixed. Many acceptability items met the feasibility criteria of 75% of participants rating moderate or higher, including items related to getting started with the smartband, wearing the smartband and being notified of smoking. However, acceptability ratings for the mindfulness exercises were more mixed, including low ratings of liking and moderate ratings of helpfulness. Interestingly, mindfulness exercises were rated as helpful in the moment, immediately after completing the exercise, whereas retrospective ratings of helpfulness were closer to ‘undecided’, which was the midpoint for these items. Likewise, retrospective ratings of whether RAIN helped with craving were closer to ‘undecided’, despite in the moment craving ratings decreasing with RAIN. (Note that no other acceptability items had ‘undecided’ as the midpoint, which was typically ‘moderate’, e.g. ‘moderately agree’). These findings highlight the utility of ratings collected in real time (i.e. ecological momentary assessment) to capture in-the-moment effects of brief quit smoking interventions.

Acceptability of the approach was supported by standardized measures including participants reporting a low burden on the User Burden Scale, high perceived impact on smoking on the MARS (Section F) and high acceptability, appropriateness and feasibility on the AIM-IAM-FIM. These findings allow comparison with other quit smoking interventions, including digital and other formats, using the same standardized measures. For example, the MARS was found to be the most common measure for evaluating smartphone apps for health behavior change [64].

Findings from this trial are in line with the only two related studies that have been conducted using wrist sensors to deliver smoking cessation treatment. A two-arm feasibility trial among daily smokers with HIV (*N* = 40) compared smoking cessation counseling and NRT with and without a smartwatch that detected smoking and delivered intervention content *via* a corresponding smartphone app [29]. Feasibility measures for the eight-week study included rates of recruitment, enrollment and retention and compliance and dosing of using the smartphone app and most measures met feasibility criteria. As noted above, day/hours of smartwatch use were not reported, preventing a more direct comparison with the current trial, although compliance with using the smartphone app was high. It was also not well described how smartwatch data were used to predict craving or smoking and deliver intervention content, or how prediction accuracy was evaluated. Another single arm feasibility study among daily smokers (*N* = 43) tested a just-in-time adaptive intervention with brief counseling and NRT plus a sensor suite with two wrist sensors to detect negative affect and smoking and deliver mindfulness and motivational strategies [37]. Feasibility measures for the six-week study included retention and adherence, ease of technology use and acceptability. Similar to the current trial, feasibility was mixed, acceptability was high, and it was suggested that improved technology would decrease participant burden and better detect key smoking intervention moments. Although studies using smartwatches/bands to intervene in smoking are limited, additional exciting work is underway [65–67].

## Strengths & limitations

Overall, this feasibility trial tested a highly novel approach using a smartband to detect and intervene in cigarette smoking. The study smartband is one of very few available for smoking research and is also compatible with existing smartwatches. The trial was fully remote, thereby providing additional feasibility information on ease of technology use with remote onboarding and had a fairly large sample size, providing information on scalability. Despite these strengths, the findings should be interpreted with respect to several study limitations. First, the combination of smartband, smartphone app and text message led to a complex intervention that impacted data quality, like other studies testing wearables for smoking [37]. For example, reported difficulties pairing between smartband and smartphone may have impacted treatment delivery. Likewise, despite daily monitoring of participant adherence including actively engaging participants with low adherence (e.g. sending text message reminders to wear the smartband), as well as providing ongoing technical support, participant adherence remained a challenge. Future studies will focus on reducing technological burden and issues and improving participant adherence. Next, as in earlier studies using national online recruitment for digital interventions [10,11] the study sample was largely white non-Hispanic and greater than high school educated, although we were able to enroll equal numbers by sex/gender. This study attempted to reduce known barriers to minority participation in smoking clinical trials by limiting exclusion criteria and offering fully remote treatment. Yet, despite advantages, fully remote studies need to address the ‘digital divide’ to reach those with limited internet access [68]. The feasibility of enrollment (i.e. *via* online advertising) to reach minority smokers warrants specific testing.

## Future directions

This trial demonstrated the feasibility of using a smartband to detect smoking and deliver interventions triggered by smoking or targeted to predicted smoking. Although the approach was found to be generally feasible, adherence to the intervention components (i.e. mindfulness exercises) was low. Future studies might pair the smartband with a full evidence-based smoking cessation intervention. For example, a combined behavioral intervention and medication might improve smoking outcomes, as in other wearables studies that additionally provided quit smoking counseling and NRT [28,29]. Relatedly, in light of our preliminary finding that greater adherence to mindfulness exercises was associated with better smoking outcomes, future studies could pair the smartband with a more comprehensive mindfulness-based intervention for smoking. Finally, further work is needed to predict craving or smoking based on more complete smartband data, beyond the basic timeseries data used here.

## Conclusions

This feasibility trial represents a novel method in digital interventions for smoking cessation by using a smartband to monitor and detect smoking and deliver real time brief mindfulness interventions to smartband-detected smoking events and predicted smoking events. Overall, feasibility was mixed, with high treatment fidelity, mixed adherence and acceptability and high retention of treatment starters. A main challenge was participant adherence, which could be improved by reducing participant burden and technological issues.

## Supplementary Material

Supplemental Material

## Data Availability

The data that support the findings of this study are available from the corresponding author, KAG, upon reasonable request.

## Trial registration

ClinicalTrials.gov Identifier NCT03995225; https://classic.clinicaltrials.gov/ct2/show/NCT03995225.

## International registered report identifier (IRRID)

DERR1-10.2196/32521.
