# Supplementary material for: Smartband-based smoking detection and real-time brief mindfulness intervention: findings from a feasibility clinical trial
Source: Ann Med. 2024 Jun 1;56(1):2352803. doi: 10.1080/07853890.2024.2352803 (PMC11146247; doi:10.1080/07853890.2024.2352803)
Supplement: Supplemental Material [file IANN_A_2352803_SM4571.docx]

**Supplement to Horvath, et al., Smartband-based smoking detection and real-time brief mindfulness intervention: findings from a feasibility clinical trial**

**Participant characteristics**

To inform future studies, we examined participant characteristics of those lost during enrollment. This information corresponds with the CONSORT diagram (Figure 1). There were n=1736 who accessed the screening survey, of whom n=607 completed the screening survey, n=156 did not meet inclusion criteria (n=18 had a smartphone type other than iPhone or Android), and n=451 were eligible and all provided online informed consent. Of these, n=241 confirmed their intent to participate in the study by text message. We added this step based on pilot work and previous studies of fully remote interventions in which there was large drop off between online consent and starting treatment. Of these, n=155 completed the zoom onboarding session and were considered the intent to treat sample. The n=86 individuals who intended to participate but did not complete onboarding were either lost to contact after confirming enrollment (n=45), no show to their onboarding appointment (n=28), had technical issues with the smartband that prevented participation (n=7), or had issues receiving the smartband by mail. The demographics of this group (n=86) who confirmed enrollment but did not successfully onboard were mean age 48.5±11.0 [SD] yrs.; 19/3±10.2 cigarettes per day; and race/ethnicity, n=59 (68.6%) non-Hispanic white, n=7 (8.1%) Hispanic white, n=7 (8.1%) Black or African American, n=1 (1.2%) Asian, n=12 (14%) some other race or multiracial.

Additionally, for the intent to treat sample, we measured internet health literacy using the eHealth Literacy Scale (eHEALS), which consists of eight items that define the scope of knowledge and trust with respect to perceived skills to find, evaluate, and apply electronic health information to address health-related concerns (Norman, 2006). E-health literacy is measured on a 5-point scale ranging from 1 (strongly disagree) to 5 (strongly agree). The overall range of the eHEALS score varies from 8 to 40, with a higher score indicating higher internet health literacy. In this trial, eHEALS mean=30.2±6.3 (SD), range 8-40 for the ITT sample, indicating relatively high internet literacy.

**Results from the full intent to treat sample**

The manuscript reported findings from a modified intent to treat (ITT) sample of individuals who confirmed enrollment in the trial and wore the smartband for at least one day (n=115). Findings here are reported for the full ITT sample (n=155), for whom retention was n=128 (82.6%) at 21 days, n=109 (70.3%) at 28 days, and n=98 (63.2%) at end of treatment. The full ITT sample (n=155) did not have adequate smartband use (i.e., <one day) for most feasibility measures including all treatment fidelity and adherence measures, and most acceptability measures (i.e., ratings about mindfulness exercises were not relevant because they did not receive any mindfulness exercises, which were sent based on smartband-detected smoking).

Acceptability measures deemed relevant for the full ITT sample were as follows. At onboarding (n=125), 98% of participants reported that the instructions for getting started with their smartband were easy to follow. At 60 days end of treatment, participants (n=98) reported a very low score on the User Burden Scale total score, mean = 8.03±8.18 [SD] out of a possible 60, range=0-42; subscales: difficulty of use, mean = 3.04 ± 2.72; time and social, mean = 2.63 ± 3.26; mental and emotional, mean = 1.40 ± 2.49; privacy, mean = 0.96 ± 1.63). They reported a high perceived impact on smoking behavior on the MARS (mean=4.06±0.69). Finally, they reported high acceptability, appropriateness, and feasibility on the AIM (mean=3.81±0.94), IAM (mean=3.83±0.91), and FIM (mean=3.98±0.77).

For the full ITT, average CPD decreased from baseline (18.6±8.7) to 60 days (F(1, 154)=58.7, P<.0001; LS mean change=-9.1±1.2[SEM]). One-week point-prevalence abstinence was reported by n=15 (9.9% with missing data coded as smoking). No additional participants participated in biochemical verification of abstinence. Prolonged abstinence with lapses was reported by n=32 (20.7% with missing data coded as smoking). There was a decrease in FTND from baseline (5.3±2.2) to 60 days (F(1, 155)=64.7, P<.0001; LS mean change=-1.6±0.2[SEM]); an increase in MNWS from baseline (1.3±0.7) to 60 days (F(1, 155)=153.0, P<.0001; LS mean change=0.84±0.07[SEM]); and an increase in FFMQ from baseline (79.7±10.1) to 60 days (F(1, 153)=10.5, P=0.002; LS mean change=3.0±0.9[SEM]).

**Descriptive report of exit interviews**

Exit interviews were conducted with n=10 randomly selected participants on video chat and included the open-ended questions “What did you find helpful or not helpful about [study component]?” and “How did [study component] impact or not impact your smoking?” for the study components: 1) the overall study, 2) wearing the smartband, 3) being notified about your smoking, 4) smartphone app, 5) online mindfulness training, 6) mindful smoking exercise, 7) RAIN exercise. Participants were also asked, “What would you change about the study?” Participants received $10 for completing the exit interview.

For 1) the overall study, the smartband app and smoking notifications were reported as helpful, and smartband technical issues and RAIN notifications being sent to often were reported as unhelpful. 2) Wearing the smartband was reported to help participants be mindful/conscious/aware of smoking, although one participant reported that they would have liked to wear the smartband on both hands. 3) Smoking notifications were reported to help participants be mindful/conscious/aware of their smoking, smoking habits, and quit smoking goals. 4) Almost all participants (n=9) reported that the smartband app was helpful for tracking their smoking. However, technical issues were reported including that the smartband did not detect their smoking or did not detect that they wore the smartband all day. 5) The online mindfulness training was described by all participants as helpful and clear, with nothing reported as unhelpful. 6) For mindful smoking, most participants (n=8) reported that the mindful smoking exercise helped them to bring attention to their smoking, think about what they were really getting from smoking, and even start to dislike smoking. Individual participants reported that the mindful smoking exercise was inconvenient (they wanted to do something else while smoking) or that they didn’t have internet service to complete the exercise. 7) For RAIN, most participants (n=8) reported that the RAIN exercise was helpful, however most (n=6) also reported receiving too many RAIN exercises, and individual participants reported that RAIN made them want to smoke or increased their craving. Finally, participants reported that their smoking was impacted by tracking smoking and using the mindfulness exercises, with most (n=8) reporting that these study components helped them to reduce their smoking, and others reporting that these aspects helped bring attention to their smoking and habits. Changes suggested for the study by individual participants included: fix smartband technical issues, fix timing and reduce number of RAIN exercises, include a variety of exercises, include exercises that take your mind off smoking or have humor/fun, make the study longer to provide more support, and provide initial information on the negative effects of smoking.

**Table S1. Participant flow**

Procedures Day 0 21 28 60

Smartband smoking detection and notification X X X X

Mindful smoking X X X

Online mindfulness training X X

RAIN X X

Primary outcome measures

Survey X X X X

Adherence X X X X

Mindfulness exercise ratings X X X
